# MicroRNAs Regulate Multiple Aspects of Locomotor Behavior in *Drosophila*

**DOI:** 10.1534/g3.119.400793

**Published:** 2019-11-06

**Authors:** Nathan C. Donelson, Richa Dixit, Israel Pichardo-Casas, Eva Y. Chiu, Robert T. Ohman, Justin B. Slawson, Mason Klein, Tudor A. Fulga, David Van Vactor, Leslie C. Griffith

**Affiliations:** * Department of Biology and Volen National Center for Complex Systems, Brandeis University, Waltham, MA 02454,; † Department of Cell Biology and Program in Neuroscience, Harvard Medical School, Boston, MA 02115, and; ‡ Department of Physics, Harvard University, Cambridge, MA 02138

**Keywords:** microRNA, motor behavior, Drosophila melanogaster

## Abstract

Locomotion is an ancient and fundamental output of the nervous system required for animals to perform many other complex behaviors. Although the formation of motor circuits is known to be under developmental control of transcriptional mechanisms that define the fates and connectivity of the many neurons, glia and muscle constituents of these circuits, relatively little is known about the role of post-transcriptional regulation of locomotor behavior. MicroRNAs have emerged as a potentially rich source of modulators for neural development and function. In order to define the microRNAs required for normal locomotion in *Drosophila melanogaster*, we utilized a set of transgenic Gal4-dependent competitive inhibitors (microRNA sponges, or miR-SPs) to functionally assess ca. 140 high-confidence *Drosophila* microRNAs using automated quantitative movement tracking systems followed by multiparametric analysis. Using ubiquitous expression of miR-SP constructs, we identified a large number of microRNAs that modulate aspects of normal baseline adult locomotion. Addition of temperature-dependent Gal80 to identify microRNAs that act during adulthood revealed that the majority of these microRNAs play developmental roles. Comparison of ubiquitous and neural-specific miR-SP expression suggests that most of these microRNAs function within the nervous system. Parallel analyses of spontaneous locomotion in adults and in larvae also reveal that very few of the microRNAs required in the adult overlap with those that control the behavior of larval motor circuits. These screens suggest that a rich regulatory landscape underlies the formation and function of motor circuits and that many of these mechanisms are stage and/or parameter-specific.

Genetic model organisms have served as powerful platforms for dissecting the cellular and molecular logic underlying a wide variety of behaviors, providing a foundation for our understanding of the molecular underpinnings of complex human phenomena such as circadian rhythms and memory formation (Keene and Waddell 2007; Hardin 2011). Locomotion is an ancient behavior, characteristic of every metazoan species with a nervous system, and is amenable to a similar approach (Griffith 2012). Although the precise connectivity and constituents of motor circuits are not yet known in most species, the majority of organisms use common mechanisms to govern coordinated motor activity (Marder *et al.* 2005). Powerful genetic approaches in *Caenorhabditis elegans* and *Drosophila melanogaster* have been applied to the assembly of these motor circuits (Wolinsky and Way 1990; Kohsaka *et al.* 2012) but the landscape of posttranscriptional regulation of gene expression in these circuits remains largely uncharted (Menon *et al.* 2013).

Among the many classes of translational regulators, microRNAs (miRs) have emerged as a rich potential source for modulation of nervous system function (Kosik 2006; McNeill and Van Vactor 2012). These small non-coding RNAs regulate the translation and stability of mRNAs and have been implicated in many cellular processes (Bushati and Cohen 2007; Flynt and Lai 2008). MiRNAs are generated from larger precursors through a series of cleavage events mediated by the enzymes Drosha and Dicer (Du and Zamore 2005). In their mature 21-24 nucleotide form, miRNAs are loaded into the RNA-induced silencing complex (RISC) (Bartel 2009). As part of the RISC, miRNAs serve as guides for the association of target RNAs with partially complementary sequences known as miRNA response elements (MREs) often located in the 3′ untranslated region (UTR). Binding of RISC to an mRNA decreases its translation by either direct interference with template-directed protein synthesis or by causing its degradation (Guo *et al.* 2010; Bazzini *et al.* 2012). MiRNAs can regulate cellular processes by modulating the expression of gene networks carrying matching MREs. The particular suite of proteins that is regulated in a given context will depend on the presence of potential target mRNAs in the miRNA-expressing cell. Although some miRNA functions can be ascribed to particular individual target genes (Bushati and Cohen 2007; Flynt and Lai 2008), others are likely to depend on modest expression changes distributed across many targets in the network. The functional role(s) of coordinated network tuning can therefore be determined through genetic manipulation of the upstream miRNA.

A limited number of studies have been done to address the role of specific miRNAs in motor systems. Analyses of several candidate miRNAs during neuromuscular junction (NMJ) development in *Drosophila* suggest that multiple aspects of synapse architecture, remodeling and/or function are under post-transcriptional regulation (*e.g.*, (Tsurudome *et al.* 2010; Loya *et al.* 2014)), but the impact of miRNA function on larval locomotor behavior was examined for only one of these genes (Sun *et al.* 2012; Wang *et al.* 2014). Global manipulation of miRNA levels in dopaminergic cells by loss of Dicer activity affects locomotor behavior in flies (White *et al.* 2010). Similarly, reduction of Dicer in mammals causes locomotor defects in part due to problems with the development of dopaminergic neurons (Huang *et al.* 2010; Pang *et al.* 2014). This raises the questions of which specific miRNAs regulate locomotion and whether miRNA regulation of locomotion involves mainly developmental assembly of motor neural circuits or an ongoing modulation of circuit function.

Work in *C. elegans* in which miRNAs and miRNA families were mutated has suggested that a relatively small number of miRNAs are essential for locomotion in this organism (Miska *et al.* 2007; Alvarez-Saavedra and Horvitz 2010). In rodents, several studies have implicated individual miRNAs in motor behavior, for example miR-128 (Tan *et al.* 2013) and miR-9 (Haramati *et al.* 2010). These studies argue that miRNAs provide a potentially important level of regulation in motor circuits, but the use of null mutants and chronic global manipulations did not allow investigators to disambiguate developmental and functional roles or to understand the tissue-specificity of miRNA actions. Recently, using a very specific assay for early larval self-righting behavior to screen many deletion alleles, a surprisingly large number miRNA were shown to modulate various aspects of this simple patterned motor response (Picao-Osorio *et al*. 2017). Interestingly, multiple miRNA hits in this focused screen appear to converge on the developmental patterning gene Abdominal-B (Abd-B), a HOX-class transcription factor (Picao-Osorio *et al*. 2017), suggesting that developmental events are a major target of translational regulation.

To further address the role of miRNAs in general baseline locomotor behavior we carried out a set of simple genetic screens using a library of so-called miRNA “sponge” (miR-SP) transgenic lines designed to inhibit the function of ca. 140 high confidence miRNAs (Fulga *et al.* 2015). MiRSPs offer the ability to dissect the role of individual miRNAs with both spatial and temporal precision (Ebert and Sharp 2010; McNeill and Van Vactor 2012). The efficacy of this technique has been demonstrated in a number of studies in *Drosophila* and other species (Loya *et al.* 2009; Bejarano *et al.* 2012; Pathania *et al.* 2012; Li *et al.* 2013; Loya *et al.* 2014; Lu *et al.* 2014; Busto *et al.* 2015; Fulga *et al.* 2015; Mugat *et al.* 2015; Goodwin *et al.* 2018). More comprehensive comparison of adult viability phenotypes detected using our expanded toolkit of miR-SPs (Fulga *et al.* 2015) with data from similar screens of null alleles (Chen *et al.* 2014) also shows that lethal phenotypes are rare and that miR-SPs recapitulate the vast majority of null phenotypes (Fulga *et al.* 2015). The very low frequency of major developmental defects in external body plan or muscle tissue detected with the miR-SP collection made screens for behavioral phenotypes feasible (Fulga *et al.* 2015).

Our aim for the current study was to capture the broadest set of potential regulators using *ubiquitous expression*, and then to explore the underlying temporal and spatial logic of their function using more selective expression. Since the adult and larval stages are fundamentally different in body plan and locomotor strategy, we screened both second instar larvae and mature adults. For miR-SP lines that gave adult phenotypes, we used *Gal80^ts^* to determine which miRNAs were acting to regulate development of adult circuits and which were required for ongoing adult function. These screens yielded a surprisingly large number of miRNAs that modulate different aspects of spontaneous baseline locomotion. Most appeared to function within the nervous system and to affect developmental processes. The majority of miRNAs that had exclusively adult function displayed phenotypes which suggested involvement of the miRNA in specific locomotor parameters as opposed to global regulation of behavior. Our analysis provides important new information on the complexity of post-transcriptional regulation in the development and function of motor circuits. Understanding the role of miRNAs in locomotor circuits will also be critical for the interpretation of miRNA phenotypes in other behaviors that may have motor outputs. Thus, we present this data as a resource for others who hope to use behavioral approaches to map miRNA functions in the future.

## Materials and Methods

### Adult fly husbandry

For adult behavioral studies *tubulin-Gal4* (*tub-Gal4*; a gift from Norbert Perrimon), *C155-Gal4* (first chromosome enhancer trap insertion of Gal4 into the *elav* locus; (Lin and Goodman 1994) and *tubulin-GAL80^ts^* (*tub-GAL80^ts^*; (Mcguire *et al.* 2003) were used to drive UAS-miR-SP. Male UAS-miR-SP males were crossed with virgin females from the specific *Gal4* strains to create each population of adults. The crosses were fed a dextrose/cornmeal fly food media and kept in vials within a 25° incubator with a 12-hour light/12-hour dark cycle, except for crosses with *tub-Gal4*;*tub-Gal80^ts^*, which were raised at 18°. All F1 male progeny were collected upon eclosion and allowed to age communally for 4-6 days before behavioral trials. The *tub-Gal4;tub-GAL80^ts^* progeny males were transferred to 29° upon eclosion to inhibit the *tub-Gal80^ts^* and allow for several days of *Gal4* activity.

### Larval husbandry

For larval behavioral assays, *elav-Gal4* (Bloomington Stock #8760)(Berger *et al.* 2007), a chromosome 3 transgene, was used to drive UAS-miR-SP. To improve phenotype penetrance for acute expression of *miR-SPs* a mutation in *Dicer-1* (*Dcr-1^1D14^*) was recombined onto the *elav-Gal4* chromosome to generate an *elav-Gal4*, *Dcr-1*^*1D14*^/balancer line. This line was crossed to homozygous UAS-miR-SP lines and mCherry-positive progeny used for assays. The use of a heterozygous *Dcr-1* background weakens the RISC system, enhancing the effects of miR knockdown, a phenomenon we noted while carrying out the original screens (Fulga *et al.* 2015).

### Sponge library

To provide an unbiased screening tool, a library of miR-SPs addressing the full collection of high confidence *Drosophila* miRs was constructed (Loya *et al.* 2009; Fulga *et al.* 2015). Each miR-SP line contains repeats of sequence complementary to an individual *Drosophila* miRNA which can be expressed under control of the Gal4/UAS system (Brand and Perrimon 1993). All miR-SP constructs were designed in the Van Vactor lab using an integrase mediated chromosomal insertion of a cassette carrying twenty repeated miRNA seed sequence complements with unique spacers downstream of a promotor with ten Gal4-UAS (upstream activating sequence) repeats and an mCherry expression reporter, and flanked by gypsy insulator sequences (Fulga *et al.* 2015). Each sequence was designed with mismatches at positions 9-12, the position normally cleaved by Ago2, allowing the miR-SP to stably inhibit function of the cognate miR (Ebert *et al.* 2007). The miR+linker sequences were checked against every mature fly miR in the database to avoid off-target effects. A concatamer of “Scrambled-SP” sequence inserts that show no overlap with any known miRNA species in *Drosophila* was created to provide a genetically matched control for the miR-SP cassette (Loya *et al.* 2009). To maximize miR-SP cassette expression for initial screening, double insert (2x) lines were created carrying cassette insertions in both Attp2 and Attp40 sites on chromosomes 2 and 3, respectively (Fulga *et al.* 2015).

### Adult locomotor protocol

To reduce variability, recording sessions were performed under consistent temperature, humidity and illumination conditions. Experiments were conducted within an environmentally controlled room (25°, 70% relative humidity) within the same 2-h window each day. Using mouth aspiration, single male flies were loaded into (40 × 10 mm) petri dish cover arenas lined and secured with Parafilm. Each chamber contained one fly. This allowed for a light-transmissive enclosure that was isolated from other external cues. Flies were allowed to freely explore their arenas for 5 min while being observed and recorded by video camera and tracking software at a 10 Hz resolution – see (Donelson *et al.* 2012) for software description. N = 36 flies per genotype. Scramble control flies were run every day to control for day-to-day variation.

### Adult analysis and line exclusion

The data files were processed and analyzed using custom-made software as described previously (Slawson *et al.* 2011). Locomotor values for each fly were obtained for the following variables: average speed, average acceleration, total distance, percentage active, number of active bouts, and active bout length. Since no single univariate test could definitively identify a miR-SP as being locomotor deficient, we reduced the number of variables to the four (average speed, average acceleration, distance, and number of active bouts) that accounted for the greatest significant separation of the miR-SP and Scramble using a multivariate analysis of variance (MANOVA). Lines that were not significantly different from Scramble were excluded from further analysis. We then took the significantly different lines and analyzed them further using a Dunnet’s Test for each independent variable to find out where the deficiency was for each miR-SP compared to control. For continued inclusion, a line had to be significantly different from Scramble-SP for at least one locomotor variable. This analysis was necessarily conservative, as the initial MANOVA identified a large cohort of lines that were significantly different due to the combined effect of multiple parameters, yet failed to meet significance under a single univariate test. Thus, we have high confidence in phenotypes for the remaining lines. Finally, lines were grouped according to the type of locomotor deficiency: (1) those dealing with how active a fly was (percentage active, number of active bouts, active bout length), or (2) those characteristics that relate to how far a fly traveled (distance, speed, acceleration). The phenotypes induced by miR-SP expression were sorted into the “active”, “distance”, or “both” categories (see Discussion).

### Larval locomotor behavior assay and tracking software

For each larval genotype, 10-to-20 adult female *elav-Gal4*, Dcr-1^*1D14*^/+ flies were placed into embryo collection cages to mate adult miR-SP transgenic males (2:1) over a grape juice-supplemented agar 60 × 15 mm plate, and a droplet of re-hydrated yeast for 2-4 h at 25° and 80–90% relative humidity. Collection plates with fertilized eggs were incubated for 72-80 h under the same conditions. 2^nd^ instar larvae, identified by anterior and posterior spiracle and mouth hook morphology, were gently washed with 1X phosphate saline buffer and placed onto dry agar plates for mCherry expression screening; GenII miR-SPs were designed with mCherry as a reporter of SP cassette expression (Fulga *et al.* 2015). Between 10-to-20 larvae were from each cross were transferred to a 245 × 245 × 18 mm 1% Bacto-agar petri dish (BD Biosciences) for locomotion analysis. Individual larvae were carefully placed on the center of the agar plate but separate from each other to minimize collisions among individuals from start. Locomotion was recorded for 200 s at room temperature with a 5 MP CMOS camera at 20 fps (Mightex Systems, Toronto, Canada) using a 12.5 mm lens (Fujifilm, Tokyo, Japan). Recording area was illuminated with IR LEDs to maximize contrast and to minimize external light cues. Data capture and extraction from each tracked larva was run in a custom-designed Labview program (National Instruments, TX, USA) previously described (Kane *et al.* 2013). Data included the position of the center of mass, the outline of the body, position of the head, tail, and midline running down the center of the larva. Runs were defined as periods of forward movement with the head aligned with the body; turns were defined as periods of slow or no forward movement accompanied by body bends (head-sweeps); and pauses were defined as periods of slow or no forward movement in which the head remained aligned with the body. Initial analysis was carried out in MATLAB (Mathworks, MA, USA) and then further processed as outlined above and in Gershow *et al.* (Gershow *et al.* 2012). MatLab scripts are available at: https://github.com/masonklein/LarvaLocomotion/.

### Data availability

All sponge lines are available at the Bloomington Stock center (see Reagent Table for all lines uploaded to GSA figshare portal). The authors affirm that all data necessary for confirming the conclusions of the article are present within the article, figures, and supplemental tables. Statistical data for behavioral assays was uploaded to GSA figshare portal. Supplemental material available at figshare: https://doi.org/10.25387/g3.9642143.

## Results

In order to define the extent and complexity of miRNA regulation for locomotor behavior, we needed a genetic method to achieve loss-of-function (LOF) individually for many miRNA genes. Having recently demonstrated the efficacy of competitive inhibition using miR-SP technology (Loya *et al.* 2009; Ebert and Sharp 2010), we employed a collection of second-generation miR-SP transgenic strains designed to inhibit 140 high-confidence miRNAs in *Drosophil*a (Fulga *et al.* 2015) (see Materials and Methods). The miR-SPs and Scramble-SP controls were generated via integrase-mediated insertion into defined loci on the second and third chromosomes using the same acceptor strain thus creating a consistent genetic background for behavioral assays (Fulga *et al.* 2015). Importantly, the conditional LOF induced by miR-SP also made it possible to examine both the spatial and temporal selectivity of miRNA function. Additionally, because miR-SPs typically generate a partial LOF whose dosage can be controlled in time and space (Loya *et al.* 2009), they can be used to examine the roles of miRNA genes for which null mutations are lethal or reveal early phenotypes that might mask a later function.

**Figure 1:**
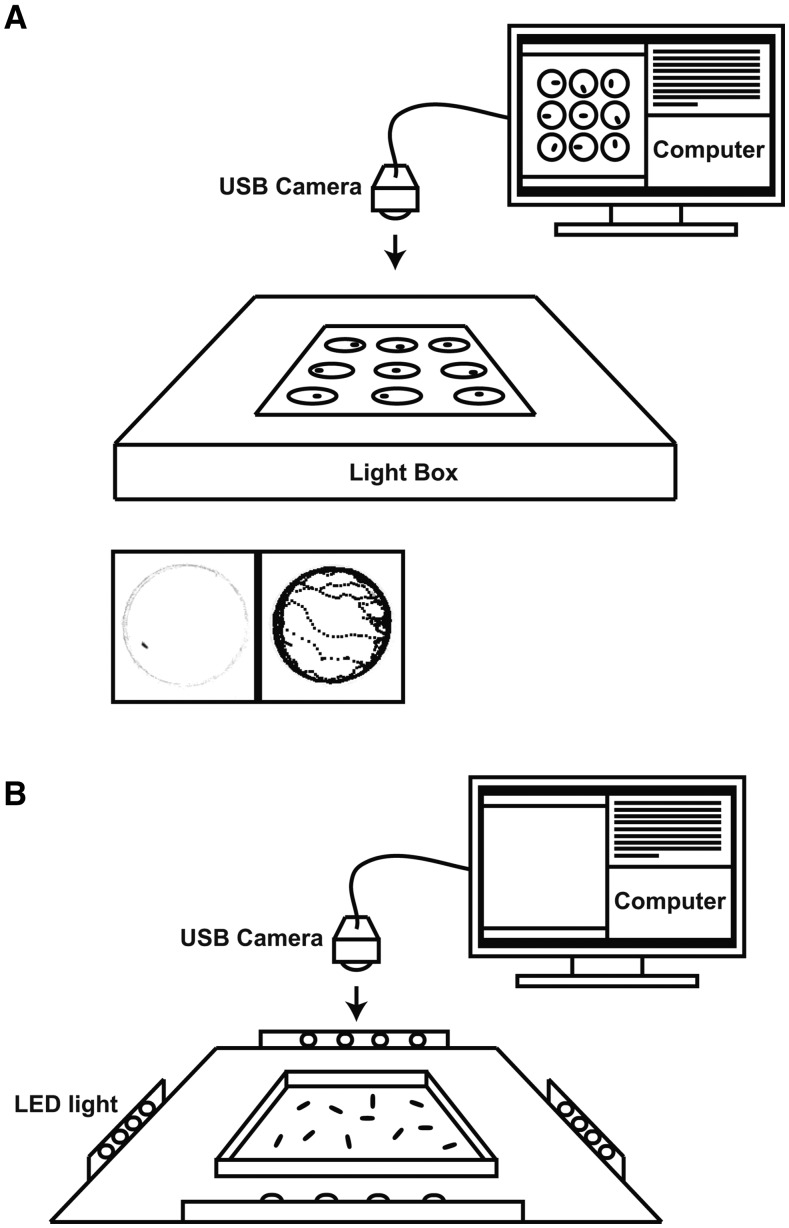
Locomotor Tracking Assays and System Controls. Flies/larval locations were recorded via acquisition software using top-down video tracking. (a) Adult platform. Underlighting illumination provided high contrast for tracking 9 flies at a time, placed individually into circular arenas. (b) Larval platform. LED arrays were used for side illumination for 25-50 larvae to be tracked simultaneously within a single arena.

Because miRNAs often function to tune the expression of downstream genes within narrow limits (Flynt and Lai 2008; McNeill and Van Vactor 2012), we required behavioral assays with statistical power capable of detecting even subtle quantitative changes in locomotion. In order to classify individual phenotypes, we also needed multiparametric analysis that could detect independent modulation of distinct aspects of the behavioral output. To measure adult behavior, we utilized an automated video recording platform where spontaneous locomotion is recorded simultaneously for individual animals in nine separate arenas over a five-minute period (Figure 1A). Relative position of each animal in the arena is continuously determined by image contrast-based “Tracker” software that calculates the acceleration, speed, distance, duration and number of locomotor bouts (as described by (Donelson *et al.* 2012)). An analogous video recording arena was constructed for the analysis of larval locomotor behavior (Figure 1B; see below) using a different algorithm designed and optimized for tracking populations of animals at this early phase of the life cycle (as described in (Kane *et al.* 2013); see Materials and Methods).

### Identification of miRNAs that regulate adult locomotion

Before initiating a genetic screen of miR-SP strains, we compared baseline locomotion in the adult assay with standard wild type control strains (*Canton-S* and *w^1118^*) and the *Gal4* driver strains to assess the effects of genetic background. Our initial screen was designed to examine ubiquitous inhibition of miRNA activity using the *tub-Gal4* driver. We detected a subtle yet consistent background effect of the *tub-Gal4* driver compared to *Canton-S* wild type. However, the tub-Gal4;Scramble-SP control was not significantly different from *tub-Gal4* across multiple locomotor parameters. This highlights the importance of genetic background and indicated that the most informative “wild type” control genotype for our screen was *tub-Gal4;Scramble-SP* rather than the more standard *Canton-S* or *w^1118^*.

We therefore screened the miR-SP collection using *tub-Gal4* followed by quantitative analysis of six parameters (average speed, average acceleration, distance, and number of active bouts as dependent variables) using Multivariate Analysis of Variance (MANOVA) to identify lines that displayed abnormal locomotor behavior compared to *tub-Gal4;Scramble-SP* controls. Because some miR-SP lines display penetrant lethal phenotypes when crossed to *tub-Gal4* (Fulga *et al.* 2015) only 136 of the 141 available miR-SP lines were screened. Once hits were identified by multivariate statistics, we then performed ANOVA and other post-hoc univariate statistical analysis to allow comparisons of significant changes in each individual parameter (see Materials and Methods).

**Figure 2:**
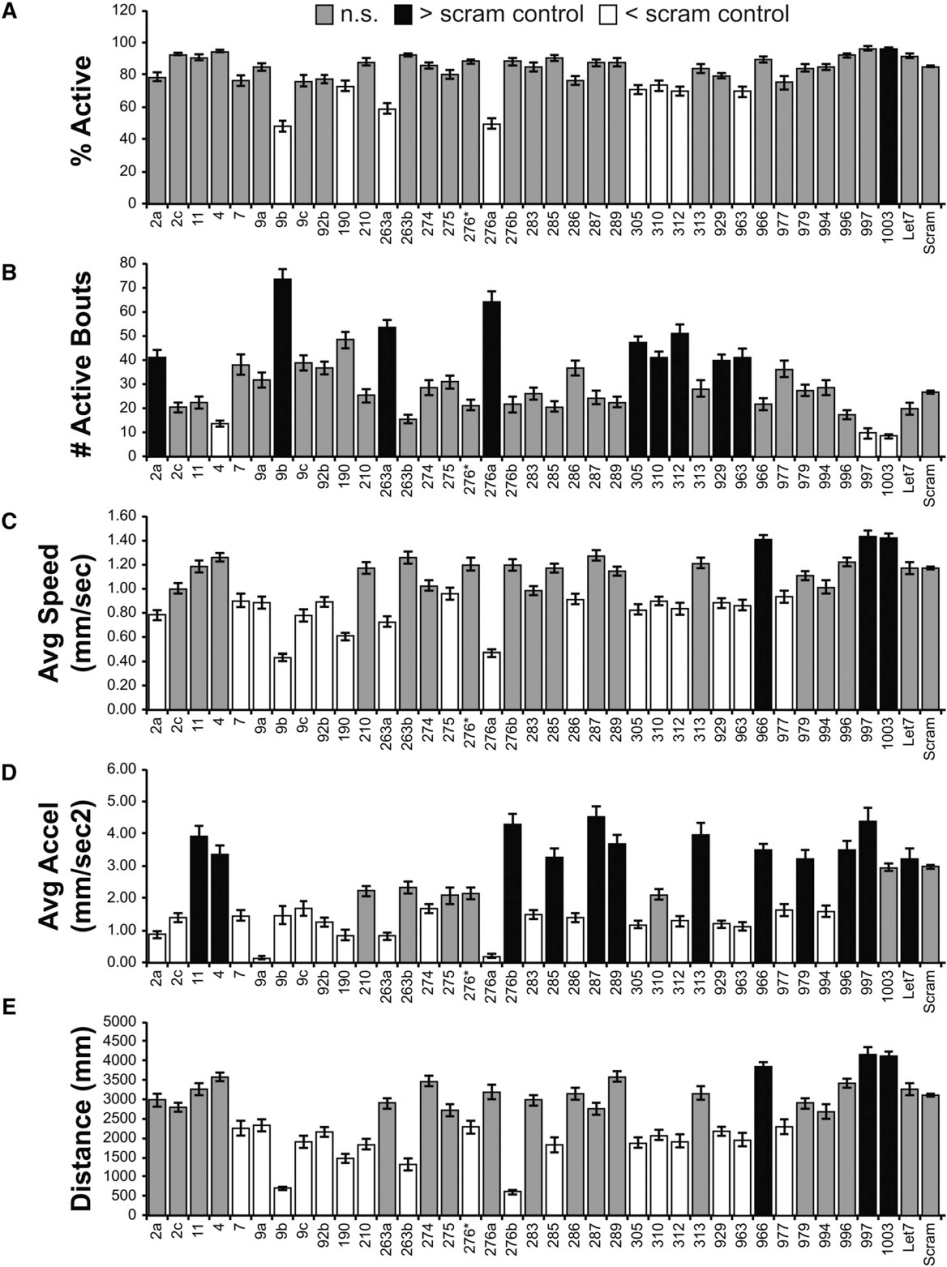
Univariate Statistical Analysis of Adult Locomotor Parameters (*tub-Gal4*). Bar plots from five locomotor parameters (%Active, #Active Bouts, Average Speed, Average Acceleration, and Distance) show how the different miR-SP lines differ from the Scramble control (Scram). Only miR-SP lines that differed in at least 1 parameter are included. Gray bars are not significantly different from Scram. Black bars are significantly greater than Scram. White bars are significantly less than Scram. Statistical significance was determined using a Dunnett’s Test, with Scram designated as control. Bars display the mean value for each line +/− the Standard Error of the Mean. All Mean, SEM, and N values are in provided in the Supplemental File.

Our initial screen identified 37 miR-SP strains with significant effects in one or more of five different parameters (Figure 2), suggesting that normal locomotion requires the activity of nearly one third (27%) of the miRNAs tested (see Supplemental Database). While the number of lines displaying phenotypes was large, comparison of effects in multiple parameters suggested underlying specificity for most of the hits in our screen. For example, only three miR-SP lines displayed significant changes in all five parameters: *miR-305SP*, *miR-312SP and miR-963SP*. Of miR-SPs that displayed selectivity to a single parameter, only one was specific to average speed (*miR-275SP*), whereas three lines were specific to distance (*miR-210SP*, *miR-263bSP* and *miR- 276SP*). The largest category of parameter-specific phenotypes was average acceleration, which includes nine miR-SP lines (Figure 2). Seven of these nine miR-SPs elevated average acceleration, whereas two displayed reduced acceleration.

Although miR-SPs have been shown to display a varying degree of cross-competitive activity for miRNAs of closely related gene families (Bejarano *et al.* 2012; Fulga *et al.* 2015) we found the locomotor phenotypes of related miR-SPs to display distinct properties. For example, we observed non-overlapping phenotypes for three K-box family miRNAs (miR-2a/2c/11), suggesting some degree of functional specialization (Figure 2). Within the miR-9 family, we found comparable phenotypes for *miR-9aSP* and *miR-9cSP* in decreasing average speed, acceleration and distance. However, *miR-9bSP*, induced a very dramatic increase in the number of active bouts and a decrease in the percentage of active adults that was not observed for *miR-9aSP* and *miR-9cSP* (Figure 2). Similarly, within the miR-92/310-313 family, we found overlapping effects for *miR-92bSP*, *miR-310SP* and *miR-312SP*; however, the overlap did not extend to all parameters (Figure 2). These results suggest that while there may be target overlap, differences in expression patterns of miR family members could potentially lead to unique behavioral roles.

### Defining temporal and spatial domains of miRNA regulation of locomotion

**Figure 3:**
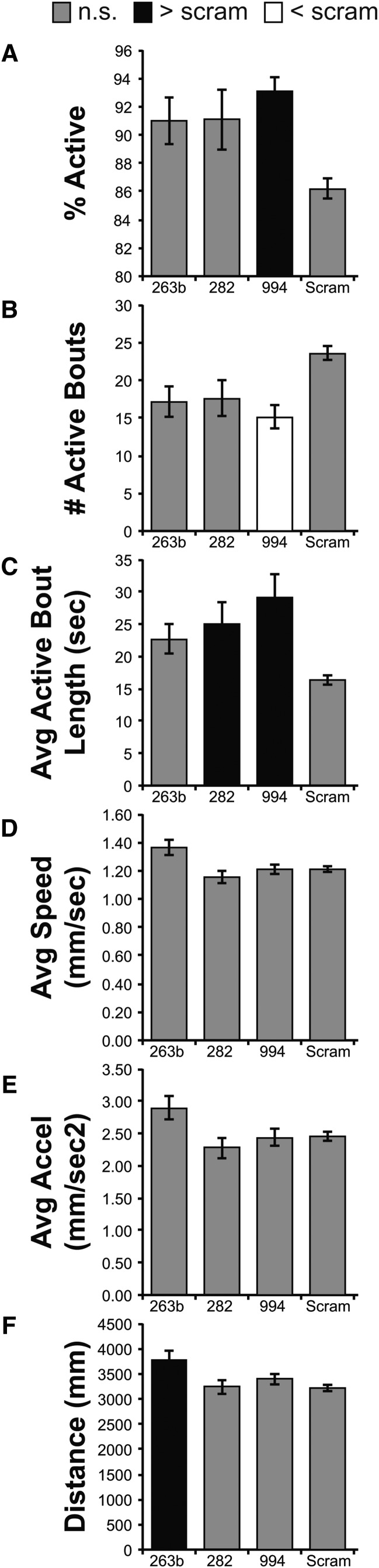
Temporal Analysis of microRNAs that Control Adult Locomotion (*tub-Gal80^ts^*). Bar plots from six locomotor parameters (%Active, #Active Bouts, Average Active Bout Length, Average Speed, Average Acceleration, and Distance) show how the different miR-SP lines differ from the Scramble control (Scram). Only miR-SP lines that differed in at least 1 parameter are included. Gray bars are not significantly different from Scram. Black bars are significantly greater than Scram. White bars are significantly less than Scram. Statistical significance was determined using a Dunnett’s Test, with Scram designated as control. Bars display the mean value for each line +/− the Standard Error of the Mean. All Mean, SEM, and N values are in provided in the Supplemental File.

One potential reason for the large number of miRNAs that appeared in our initial screen was the very broad expression of the *tubulin* promoter used to drive *Gal4* expression. In addition, the expression of *tub-Gal4* in both the developing and adult animal would be predicted to reveal both defects in the construction and specification of motor circuit architecture and function, as well as ongoing adult-specific functions required for the output of the locomotor circuitry. We addressed the question of temporal domain by combining *tub-Gal4* with a transgene encoding a temperature-sensitive mutant of the *Gal4* antagonist *Gal80* (Mcguire *et al.* 2003). Homozygous *tub-Gal4;tub-Gal80^ts^* animals were crossed to each *miR-SP (2x)* strain and grown at permissive temperature (18°) until eclosion, followed by a shift to 29° to allow *tub-Gal4* to be active for a period of several days prior to behavior assay. Of the 137 miR-SP lines tested with this regimen, only three *miR-SPs* displayed significant effects on one or more of six quantitative parameters: *miR-263bSP*, *miR-282SP*, and *miR-994SP*. Each of the three phenotypes was distinct from the phenotype obtained with *tub-Gal4* (Figure 3). Adult-specific inhibition of miR-263b increased average distance, but was not significant in any other parameter. The phenotype induced by expression of *miR-282SP* was specific to average bout length. Although inhibition of miR-994 altered multiple parameters, *miR-994SP* increased bout length and the percentage of active adults at the expense of the number of locomotor bouts (Figure 3). While the decrease in the overall number of hits may be partially due to differences in sponge levels that can be achieved with the *tub-Gal4;tub-Gal80^ts^* tool, the fact that the phenotypes we do see are qualitatively different than those obtained with *tub-Gal4* argues strongly that these three miRs have adult-specific roles that are separable from their actions in development.

Our observations suggest that ongoing miRNA regulation of adult motor circuit function relies upon a small number of distinct miRNA-dependent mechanisms. Interestingly, comparison of chronic and acute miR-SP expression for these miRNAs also revealed qualitative differences in phenotype, suggesting individual miRNAs may serve different functions at different stages in the life cycle. For example, the phenotype induced by expression of *miR-263bSP* was selective to regulation of distance traveled in both scenarios, however inhibition of *miR-263b* reduced distance when chronically applied but increased distance when acutely applied. Overall, our data suggest that the majority of miRNA functions relevant to locomotor behavior control developmental processes occurring prior to eclosion.

**Figure 4:**
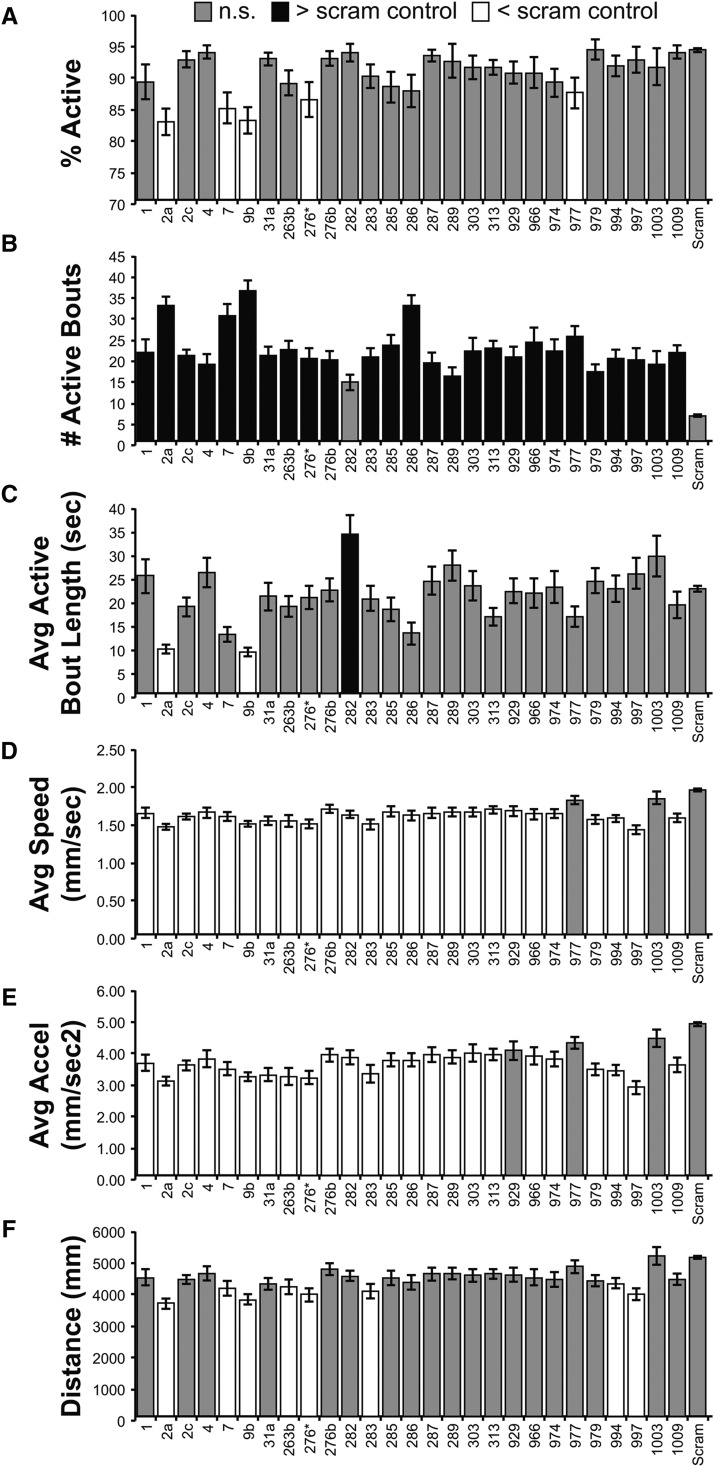
Univariate Statistical Analysis of Adult Locomotor Parameters (*C155-Gal4*). Bar plots from six locomotor parameters (%Active, #Active Bouts, Average Active Bout Length, Average Speed, Average Acceleration, and Distance) show how the different miR-SP lines differ from the Scramble control (Scram). Only miR-SP lines that differed in at least 1 parameter are included. Gray bars are not significantly different from Scram. Black bars are significantly greater than Scram. White bars are significantly less than Scram. Statistical significance was determined using a Dunnett’s Test, with Scram designated as control. Bars display the mean value for each line +/− the Standard Error of the Mean. All Mean, SEM, and N values are in provided in the Supplemental File.

Because *tub-Gal4* is expressed in many cell types in the adult and developing organism, we next asked what subset of miR-SPs might display phenotypes selective to the nervous system. Using the neural-specific driver *C155-Gal4* that is expressed at high levels during embryonic and larval development (Lin and Goodman 1994; Berger *et al.* 2007), we repeated the adult locomotor screen of miR-SP lines. In total, MANOVA analysis identified 27 miR-SP strains that displayed significant adult locomotor phenotypes when expressed with *C155-Gal4* (Figure 4). Although most of these lines also scored as significant in our *tub-Gal4* screen, several were unique, including miR-SPs inhibiting miR-1, miR-31a, miR-282, miR-303, miR-974 and miR-1009. The majority of the hits with *C155-Gal4* altered multiple parameters; only *miR-1003SP* was completely selective to the number of active bouts (Figure 4). However, comparison of phenotypes in the *tub-Gal4* and *C155-Gal4* screens using univariate statistics revealed that neural inhibition of miRNA function produced different locomotor phenotypes for many of the overlapping hits. Some of the differences were minor, and could be explained by differences in the strength of *Gal4* activity in the two drivers. For example, *miR-9bSP* shifted all parameters in the same direction in both datasets. In addition, *miR-7SP* affected distance, acceleration and speed in both datasets, but also altered the number of active bouts and the percentage of active adults when driven with the stronger *C155-Gal4* driver. However, the number of miR-SP lines that decreased both acceleration and speed was greater when expressed with *C155-Gal4*.

### miRNA regulation of larval locomotion

Diptera are holometabolous insects that undergo a profound remodeling of neural circuitry, effector organs, and body plan between larval and adult stages of the life cycle. Unlike the adult form that walks on appendages with an alternating tripod gait (Wilson 1966; Bowerman 1977), *Drosophila* larvae move by reverse peristaltic waves of segmental body wall muscles (Kohsaka *et al.* 2012). Larval muscles and NMJs are replaced during metamorphosis, raising the intriguing question of whether locomotor behaviors are regulated by distinct or overlapping sets of miRNAs in larvae and adults. To address this, we utilized 128 of the homozygous viable miR-SP strains in combination with a neural-specific *elav-Gal4* driver to analyze locomotion in second instar larvae (see Materials and Methods). We assembled a video-tracking arena illuminated with a wavelength invisible to larvae in order to record spontaneous locomotor behavior (Figure 1B). The MATLAB-based larval tracking software was optimized to extract quantitative parameters equivalent to those analyzed in our adult screen.

**Figure 5:**
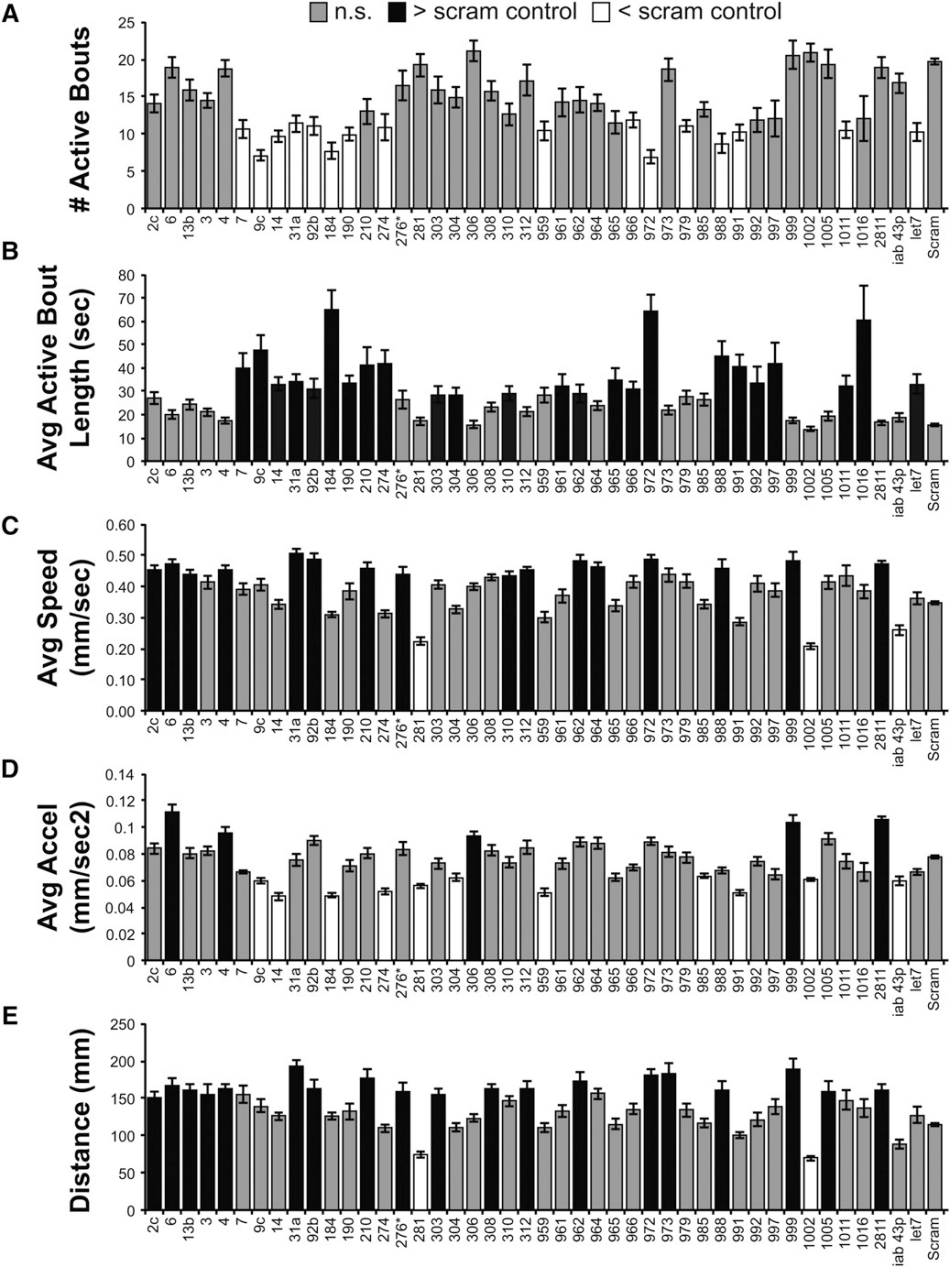
Univariate Statistical Analysis of Larval Locomotor Parameters (*elav**-Gal4*) Bar plots from five locomotor parameters (#Active Bouts, Average Active Bout Length, Average Speed, Average Acceleration, and Distance) show how the different miR-SP lines differ from the Scramble control (Scram). Only miR-SP lines that differed in at least 1 parameter are included. Gray bars are not significantly different from Scram. Black bars are significantly greater than Scram. White bars are significantly less than Scram. Statistical significance was determined using a Dunnett’s Test, with Scram designated as control. Bars display the mean value for each line +/− the Standard Error of the Mean. All Mean, SEM, and N values are in provided in the Supplemental File.

Similar to our screens of adult locomotion, a large fraction (44 of 128; 34%) of our miR-SP lines induced a significant effect on one or more locomotor parameters in L2 stage larva (Figure 5). This percentage of hits was approximately equal to the frequency of miRNA hits in an early larval deficiency screen selective to self-righting behavior (Picao-Osorio *et al*. 2017), confirming that many miRNAs play some role in establishing normal motor circuitry, but that many of the effects may not be selective to particular motor responses. Despite the large number of hits, we found larval phenotypes to exhibit a greater degree parameter selectivity than the neurally-driven miR-SPs assayed in early adulthood. In all, 14 of the hits were exclusive to one parameter. For example, inhibition of the highly-conserved miRNA let-7 only affected the number of active bouts, reducing this to nearly half of the control level (Figure 5). Nearly half of the hits (22) significantly altered the distance traveled, however, only four miRNAs appeared to be selective to this parameter: miR-3, miR-303, miR-308 and miR-1005 (Figure 5). The results were similar for average speed, with 20 miR-SPs showing significant effects, but only two being dedicated to this parameter: miR-310 and miR-964 (Figure 5). Two miRNAs were selective to average acceleration: inhibition of miR-306 elevated this parameter, whereas inhibition of miR-985 decreased acceleration. Interestingly, less than half of the miR-SPs that altered speed also effected average acceleration (7 of 20). These data suggest that different properties of the motor circuit are under control of many miRNAs, but that some miRNAs offer the means of independent modulation for one of the key parameters.

## Discussion

**Figure 6:**
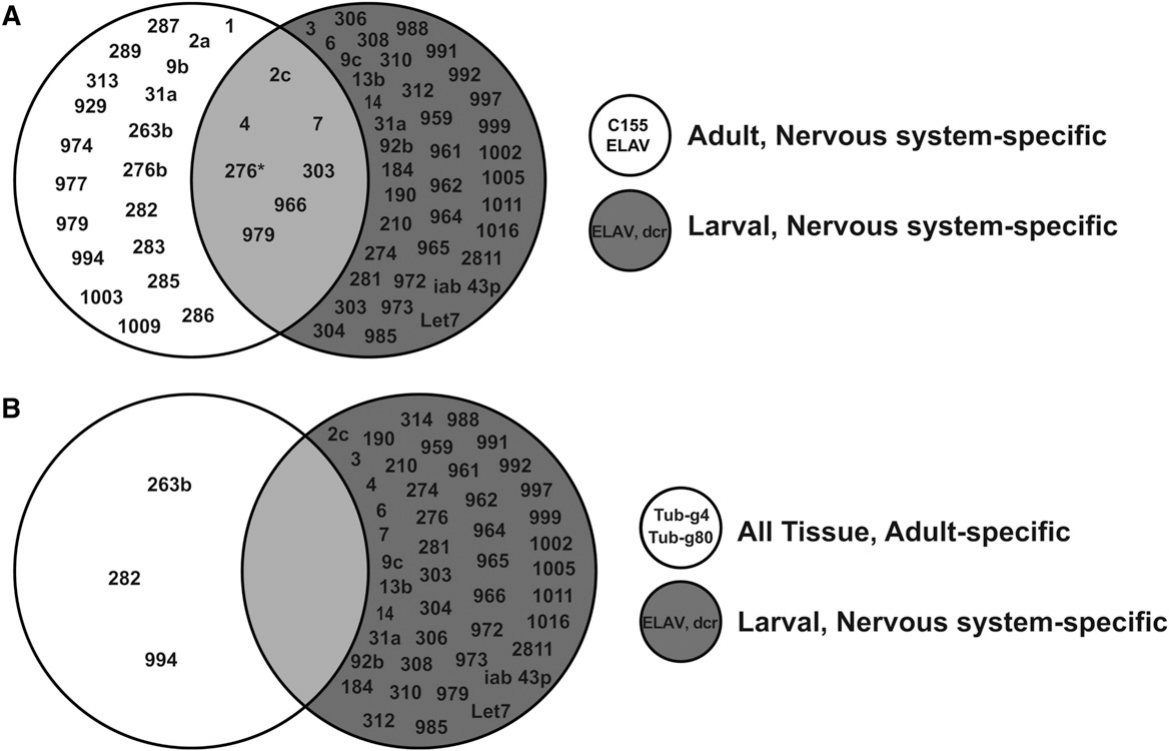
Summary of data: Venn Diagrams comparing Adult and Larval overlap. The larval and adult screens using a pan-neuronal driver (a) show that only seven of the miR-SP lines significantly affected both larval and adult locomotion indicating that the effect of miRNA is specific to life phase. Similarly, three lines are specific to adults (b) and have no effect in the larval phase.

In order to determine the complexity of miRNA-dependent regulatory mechanisms governing spontaneous locomotor behavior in *Drosophila*, we utilized conditional competitive inhibition and unbiased quantitative behavior tracking to test the function of ca. 140 miRNAs at two developmental stages. Our data suggest that a surprisingly large number of miRNAs modulate locomotion in adults and in larvae. However, comparisons between miRNAs required for normal locomotion at different developmental stages demonstrate that few miR-SPs induce phenotypes in both larvae and adults (only 8 of 66; see Figure 6A). Use of a conditional system to examine the temporal domain of miRNA function supporting normal adult locomotion reveals that only three miRNAs are required during adulthood (Figure 6B), suggesting that the majority of functions are developmental in nature. One caveat to the conclusion, however, is that we do not know if adult miR-SP levels are comparable in the temporally unrestricted and conditional situations. Interestingly, our quantitative analysis also reveals that these three adult-specific miRNAs (miR-263b, miR-282 and miR-994) display selectivity to distinct aspects of the behavioral output (Figure 3), implying that each could serve as an independent regulatory node for adult walking. Overall, our analysis suggests that post-transcriptional tuning of gene expression plays an important role in determining the baseline behavioral state, raising obvious implications for future behavioral screens dependent on locomotor output.

In contrast to previous screens of miRNAs for locomotor functions in *C. elegans* (Miska *et al.* 2007; Alvarez-Saavedra and Horvitz 2010), specific motor escape behavior in *Drosophila* larvae (Picao-Osorio *et al*. 2017), or motor-dependent behavioral defects in adult flies (Chen *et al.* 2014; Busto *et al.* 2015; Fulga *et al.* 2015), our current analysis of miR-SPs employed automated multi-parametric data processing in order to detect subtle but significant modulatory effects on distinct aspects of motor control. While spontaneous adult locomotion is notably variable when viewed in detail (Berman *et al.* 2014), we have attempted to look at higher order parameters to capture defects in overall motor coordination. Whether these gross defects reflect changes in specific stereotyped sub-behaviors is yet to be determined. The small effect sizes we see do not necessarily predict small effects in sub-behaviors, however, since there is likely to be pressure to maintain these gross parameters. Changes in multiple sub-behaviors that are compensatory would be one mechanism to achieve this.

**Figure 7:**
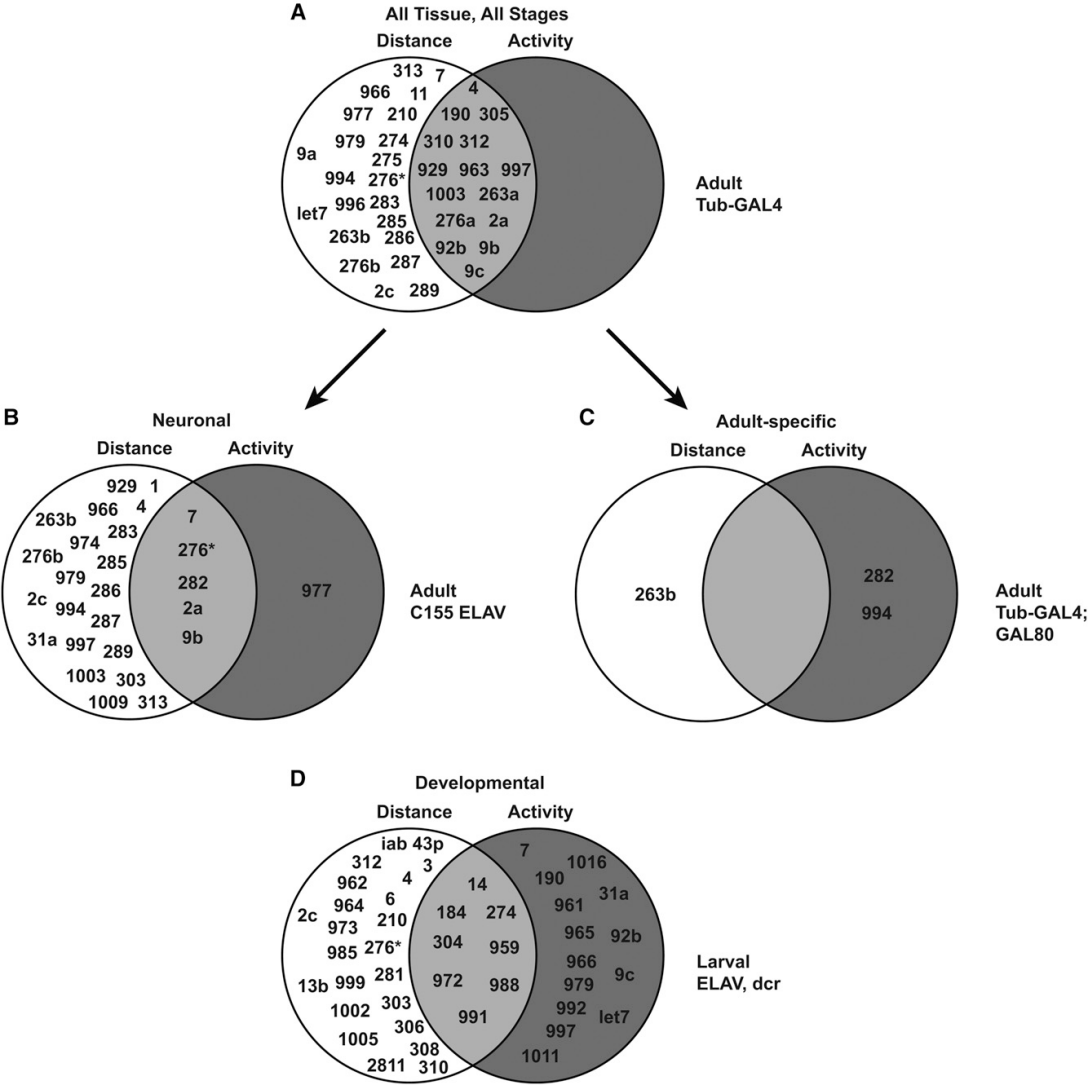
Summary of data: Venn Diagrams dividing hits into Distance *vs.* Activity. The All Tissue screen (a) used Tub-GAL4 to overexpress each miR-SP in all tissues. It shows that most of the miR-SP lines affect locomotion related to how far the fly moved rather than overall activity level of the fly. None of the lines showed an activity-only affect in adult locomotion, while twenty-two of the lines showed a distance only phenotype. This suggests that a fly can have normal rates of activity while moving significantly greater/lesser distances but not the converse. Fifteen of the lines were affected by both distance and activity variables, suggesting that it is possible to have high instances of activity, while also not moving great distances, like in miR-SP 9b. The neural (C155) screen (b) further demonstrates that distance is primarily affected. When miR-SP expression is limited only to adults (c), only three lines show significant locomotor phenotypes, suggesting that very few miRNAs are adult specific. The larval screen (d) shows a more even distribution of the miR-SP lines, with the majority of lines being either all distance affected or all activity affected.

Our multiparametric analysis of miRNAs suggests substantial functional specialization at each developmental stage. When phenotypes are grouped into effects on either overall distance traveled or frequency of locomotor activity, we find nearly all of the miRNAs required for adult locomotion affect distance while only a subset of these also modulate activity level (Figure 7A). When we repeated this analysis with neural-specific phenotypes, we found that only one of the miRNAs controlling activity was exclusive to this parameter in adults (miR-977; Figure 7B). However, when the same categories are applied to late-acting, adult-specific miRNA functions, we find that miR-263b is specific to distance, whereas miR-282 and miR-994 are specific to activity (Figure 7C).

Although spontaneous locomotion in larvae is typically continuous, larval assays indicate that most miRNAs exert some selectivity to either distance or activity (Figure 7D). Therefore, our analysis reinforces the conclusion that miRNAs control distinct aspects of motor circuit function and/or development. Interestingly, in spite of the fact that our larval screen was designed to be sensitized by reduction of Dicer activity, we found minimal overlap between miR-SP locomotor phenotypes in adult and larval stages (Figure 6A); no overlap was found for the adult-acting miRNAs (Figure 6B). Thus, while we detect many miRNA effects, they appear to be quite stage-specific. Future in depth characterization will be required to determine if the effects are mediated via changes in excitability, synaptic transmission, or the formation and/or maintenance of precise connectivity.

In addition to bringing many insights into developmental mechanisms (Collins *et al.* 2006), studies of the *Drosophila* neuromuscular junction (NMJ) have uncovered conserved signal transduction pathways and transcriptional programs that guide development and tune the ongoing activity of the circuit (Sanyal and Ramaswami 2006). Although no comprehensive *in vivo* miRNA screens for synapse development have yet been described, recent studies in *Drosophila* have identified several conserved miRNAs that regulate the development, form and/or function of neuromuscular synapses (Sokol *et al.* 2008; Loya *et al.* 2009; Tsurudome *et al.* 2010; Sun *et al.* 2012; Loya *et al.* 2014; Wang *et al.* 2014). Among these few known synaptic regulators, our current data suggest that let-7 regulates locomotor activity at both larval and adult stages (Figure 6). Notably, we did not detect a significant phenotype for *miR-124SP* even though independent *miR-124* nulls display locomotor defects (Sun *et al.* 2012; Wang *et al.* 2014); this is probably due to the hypomorphic nature of competitive inhibition (Fulga *et al.* 2015). For these reasons, it is likely that our screens represent an underestimate of the miRNAs that modulate locomotion.

Our finding that some miRNAs control distinct aspects of locomotor behavior raises the question of whether these miRNAs may be under dynamic control as a means to tune behavioral state, or whether these miRNAs specify a static baseline state of the motor circuit. Recent expression profiling identified a number of activity-responsive miRNAs in *Drosophila* larva (Nesler *et al.* 2013), including several genes that display locomotor activity in our screens. Within the set of genes down-regulated by acute potassium induced depolarization of third instar fillets (Nesler *et al.* 2013), we find that miR-304 is required in the nervous system for normal larval locomotion, whereas neural inhibition of miR-1, miR-289, and miR-304 also alters adult locomotion (Figure 7). Such miRNAs are therefore candidates to mediate adaptive responses of motor circuits, although future experiments will be required to determine what circuit and/or synaptic properties lie downstream.

It is also possible that certain activity-dependent miRNA mechanisms may be essential for the refinement of locomotor patterns. Optimal performance of motor systems appears to involve an initial experience-dependent phase at both the larval and adult stage (Hesselberg and Lehmann 2009; Fushiki *et al.* 2013). Alternatively, a subset of miRNAs may serve to support both the early development and the ongoing plasticity of neural circuits. Interestingly, a parallel screen of the miR-SP collection using an assay for intermediate term associative memory in adult animals identified a distinct set of miRs, including miR-9c, miR-31a, miR-305, miR-974 and miR-980 (Busto *et al.* 2015). However, future assays of activity-dependent plasticity will be required to determine if these miRNAs are required for experience-dependent changes in motor circuits.

The advent of genetic tools for comprehensive loss-of-function screens in *Drosophila* will offer the ability to detect miRNA roles in regulating a range of behaviors (Chen *et al.* 2014; Fulga *et al.* 2015). A recent analysis of 88 site-directed deletions of miRNA genes and gene clusters for adult climbing behavior identified nine miRNAs required for locomotion driven by negative geotaxis (Chen *et al.* 2014). Although our adult locomotor tracking assay was not dependent on geotactic stimuli, over half of the climbing-defective mutants overlap with our *tub-Gal4* screen, including miR-9a, miR-11, miR-210, miR-276b and miR-282. This is a large overlap in light of the fact that our multi-parametric data analysis was designed to detect subtle modulatory effects on distinct aspects of movement. A subsequent screen of 81 of the miRNA deletions for larval self-righting behavior in first instar larvae revealed that over 40% of these chronic and systemic mutants display defects (Picao-Osorio *et al.* 2017). While the hit rate in our screen of second instar larvae was lower (34%), likely due to the fact that miR-SP are hypomorphs, it is notable that there is substantial overlap- 40% of the hits in the Picao-Osorio screen of null mutants were hits in our miR-SP screen.

It is tempting to speculate that the large number of miRNA genes revealed by these screens may reflect distinct regulatory activities in the different classes of neurons and/or glia that contribute to the motor circuits of larva and adults. As dissection of the motor circuitry advances and Gal4 drivers become available to test each constituent cell type (Kohsaka *et al.* 2014; Itakura *et al.* 2015; Ohyama *et al.* 2015) it will be possible to address this question using conditional inhibition of miR activity.
